# Analysis of Smartphone Text Data Related to mpox from a U.S. Sample of Gay, Bisexual, and Other Men Who Have Sex with Men During the 2022 Outbreak

**DOI:** 10.1089/lgbt.2022.0307

**Published:** 2023-10-04

**Authors:** Cory J. Cascalheira, Chenglin Hong, Raiza M. Beltran, Kimmo Karkkainen, Mehrab Beikzadeh, Majid Sarrafzadeh, Ian W. Holloway

**Affiliations:** ^1^ Department of Counseling and Educational Psychology, New Mexico State University, Las Cruces, New Mexico, USA.; ^2^ Department of Social Welfare, Luskin School of Public Affairs, University of California, Los Angeles, Los Angeles, California, USA.; ^3^ Gay Sexuality and Social Policy Initiative, Luskin School of Public Affairs, University of California, Los Angeles, Los Angeles, California, USA.; ^4^ Department of Computer Science, Henry Samueli School of Engineering, University of California, Los Angeles, Los Angeles, California, USA.

**Keywords:** bisexual, gay, LGBTQ, monkeypox, mpox, social networking

## Abstract

**Purpose:**

We sought to understand technology-based communication regarding mpox (monkeypox) among gay, bisexual, and other men who have sex with men (GBMSM) during the global outbreak in 2022.

**Methods:**

Forty-four GBMSM (M_age_ = 25.3 years, 68.2% cisgender, 43.2% non-White) living in the United States participated. From May 2022 to August 2022, all text data related to mpox (174 instances) were downloaded from the smartphones of GBMSM. Text data and smartphone app usage were analyzed.

**Results:**

Content analysis revealed 10 text-based themes and 7 app categories. GBMSM primarily used search and browser, texting, and gay dating apps to share vaccine updates, seek mpox vaccination, find general mpox information, share mpox information with other GBMSM, and discuss links between mpox and gay culture. Data visualizations revealed that changes in communication themes and app usage were responsive to major milestones in the mpox outbreak.

**Conclusion:**

GBMSM used apps to facilitate a community-driven mpox response.

## Introduction

During the 2022 mpox (i.e., monkeypox) outbreak, cases were reported in >100 countries,^1^ including >30,000 cases in the United States.^2^ Mpox cases were reported predominantly among gay, bisexual, and other men who have sex with men (GBMSM),^3^ a population already disproportionately affected by the human immunodeficiency virus (HIV) epidemic and the COVID-19 pandemic.^4^ In California, for instance, ∼95% of the reported cases were among GBMSM.^5^ Public health prevention strategies included scaling up vaccination and increasing testing capacity.^6^ However, leveraging GBMSM's fluency in computer-mediated communication was an underutilized prevention strategy for mpox in particular and future infectious diseases more broadly.

GBMSM increasingly use web-based smartphone applications (e.g., social media, gay dating applications; collectively, “apps”) to connect to their social and sexual networks,^7–9^ particularly during viral outbreaks.^10^ Health mobilization through apps came to prominence during the COVID-19 pandemic as the exchange and sharing of informational resources became urgent in the absence of a comprehensive public health response.^11^ GBMSM began to increase their use of social media, texting, and dating apps (e.g., *Hornet*) to reduce infection risk^12,13^ while communicating COVID-19-specific worries and harm-reduction strategies to other GBMSM.^10^ Despite the prominence of apps in previous disease outbreaks, no study has explored how technology facilitated GBMSM's response to mpox.

The first step in designing real-time adaptive digital interventions for viral risk reduction is to understand how GBMSM use apps to communicate about public health priorities,^14^ such as mpox. Thus, this study sought to characterize how GBMSM responded to mpox by analyzing text data generated across a variety of apps. We aimed to describe major themes of GBMSM's mpox communication, understand common communication channels among GBMSM related to mpox, and explore temporal variation in major themes and communication channels.

## Methods

### Procedure

Participants were GBMSM (*N* = 44) primarily living in the metro area of Los Angeles, California. One participant was living in Indiana. They were drawn from a larger study investigating HIV risk among GBMSM. In the larger study, GBMSM were recruited through advertisements posted on social media and popular dating applications (e.g., *Grindr*). Participants were eligible for the larger study if they were over age 18 years, used Facebook and at least one gay-specific app (e.g., *Scruff*), had used a psychoactive substance in the past 3 months, had condomless anal intercourse in the past 6 months, and could read and speak English.

All participants included in this analysis had a smartphone with the Android operating system. Participants consented to install a custom-developed app that tracked their keystrokes using the Aware Framework.^15^ From May 1, 2022, to August 31, 2022, the keystroke logger saved all text (except sensitive data, such as passwords) participants typed on their smartphone across all apps (e.g., web searches, text messages, and communication on dating apps). To establish the data set on mpox, a search algorithm detected all text input using at least one of the following terms: monkey pox, monkeypox, pox, virus, viruses, orthopox, mpx, gaypox, or CDC.

The data set consisted of the participant's identification number, a time stamp, the app on which the participant entered the text, and the text itself. No identifying information was stored on study servers. The institutional review board of the senior author's institution approved this study. All participants provided informed consent.

### Data analytic plan

All descriptive analyses, comparison tests, and visualizations were performed in R version 4.1.3. Two-sided tests of proportion equality were conducted to examine statistically significant differences in proportions (GBMSM who used mpox search terms vs. those who did not). Code and data are available from the first author. To establish themes, we used conventional content analysis.^16^ The first author coded all smartphone text. To increase trustworthiness,^17^ the third author audited all themes and the entire team debriefed during group meetings.

## Results

Participants were 44 GBMSM (M_age_ = 25.3 years, standard deviation [*SD*] = 2.97) who identified as gay (68.2%), bisexual (20.5%), or as some other sexual orientation (11.4%). Over two-thirds of participants identified as cisgender (68.2%), trans men (25.0%), or nonbinary (6.8%). In terms of race/ethnicity, participants identified as White (56.8%), some other race (20.5%), Asian (11.4%), or Black (11.4%) with 84.1% stating they were non-Hispanic. Participants held bachelor's degrees (27.3%), had some college (25.0%), earned a graduate degree (22.7%), had a high school diploma or GED (13.6%), or held an associate's degree (11.4%).

A total of 21 GBMSM (47.7%) used at least one mpox term between May 1, 2022, and August 31, 2022, generating 235 unique instances of text. All 21 participants were recruited before June 4, 2022. Compared with the 23 GBMSM who did not use mpox-related terms, these 21 participants were not statistically significantly more likely to be people of color ($\chi^{2}$[1] = 1.07, *p* = 0.3), cisgender ($\chi^{2}$[1] = 0.46, *p* = 0.5), or have a college education ($\chi^{2}$[1] = 1.51, *p* = 0.2). Content analysis revealed that 74.0% of the posts were related to mpox, 20.4% to a different virus (e.g., chickenpox), 10.2% to COVID-19, and 1.3% to concerns about mpox–COVID-19 coinfection. The remaining results pertain to the 174 text instances coded as related to mpox.

As given in Table 1, content analysis revealed 10 themes related to mpox communication among GBMSM. Themes were not mutually exclusive:

**Table 1.** Illustrative Quotes of Monkeypox Themes

Theme	%	Example quote
Sharing vax update	29.9	I got my monkeypoxvaccine (28-year-old White gay man)
Seeking vax	25.3	is there a vaccine for monkeypox (27-year-old bi-racial gay nonbinary person)
Seeking general info	16.1	how many monkeypox cases in ill (18-year-old White bisexual man)
Sharing info	13.2	On a unrelated note, I have been working with a bunch of people on the internet to compile resources for those looking for possibly getting a monkeypox vaccine. If anyone is interested please DM me (28-year-old White gay man)
mpox + gay	8.1	The CDC announcement heavily centers men who have sex with men. I am not optimistic that the government will actually take this seriously since, historically, viruses impacting MSM are… Ignored, we'll say. 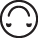 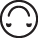 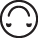 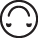 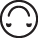 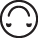 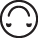 (27-year-old White gay trans man)
Seeking health info	7.5	can asymptomatic monkeypox be contagious (29-year-old Asian gay man)
Using caution	6.3	not really hahaha. been avoiding it until i got my monkeypox vax (27-year-old Asian gay man)
General mentions	6.3	monkeypox (28-year-old White gay man)
Negative experience	5.2	too busy and worried about the pox (27-year-old Asian gay man)
Using humor	4.6	Just panicked that my bacne was monkeypox until I remembered I haven't showered in like almost 2 weeks and am on T 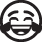 (27-year-old White bisexual trans man)

Percentage based on 174 instances of text related to mpox.

CDC, Centers for Disease Control and Prevention; DM, direct messaging; MSM, men who have sex with men; vax, vaccine.

*Sharing Vax Update*, or disclosed information about intending to get or receiving the mpox vaccine (29.9%); *Seeking Vax*, or looked for information on where and when to get the vaccine (25.3%); *Seeking General Info*, or sought general information about mpox, such as the number of cases in their local community (16.1%); *Sharing Info*, or shared facts about mpox with their social and sexual networks (13.2%); *mpox + Gay*, or made or challenged links between gay culture, same-sex behavior, and mpox (8.1%); *Seeking Health Info*, or sought health-related information, such as what mpox symptoms look like (7.5%); *Using Caution*, expressed hesitancy to engage in normal activities due to fear of contracting mpox (6.3%); *General Mentions*, or mentioned mpox generally, such as typing “monkeypox” into Google search (6.3%); *Negative Experiences*, expressed negative reactions to mpox, such as worry or frustration (5.2%); and *Using Humor*, or employing mpox-related jokes with social and sexual networks (4.6%).

**FIG. 1.**
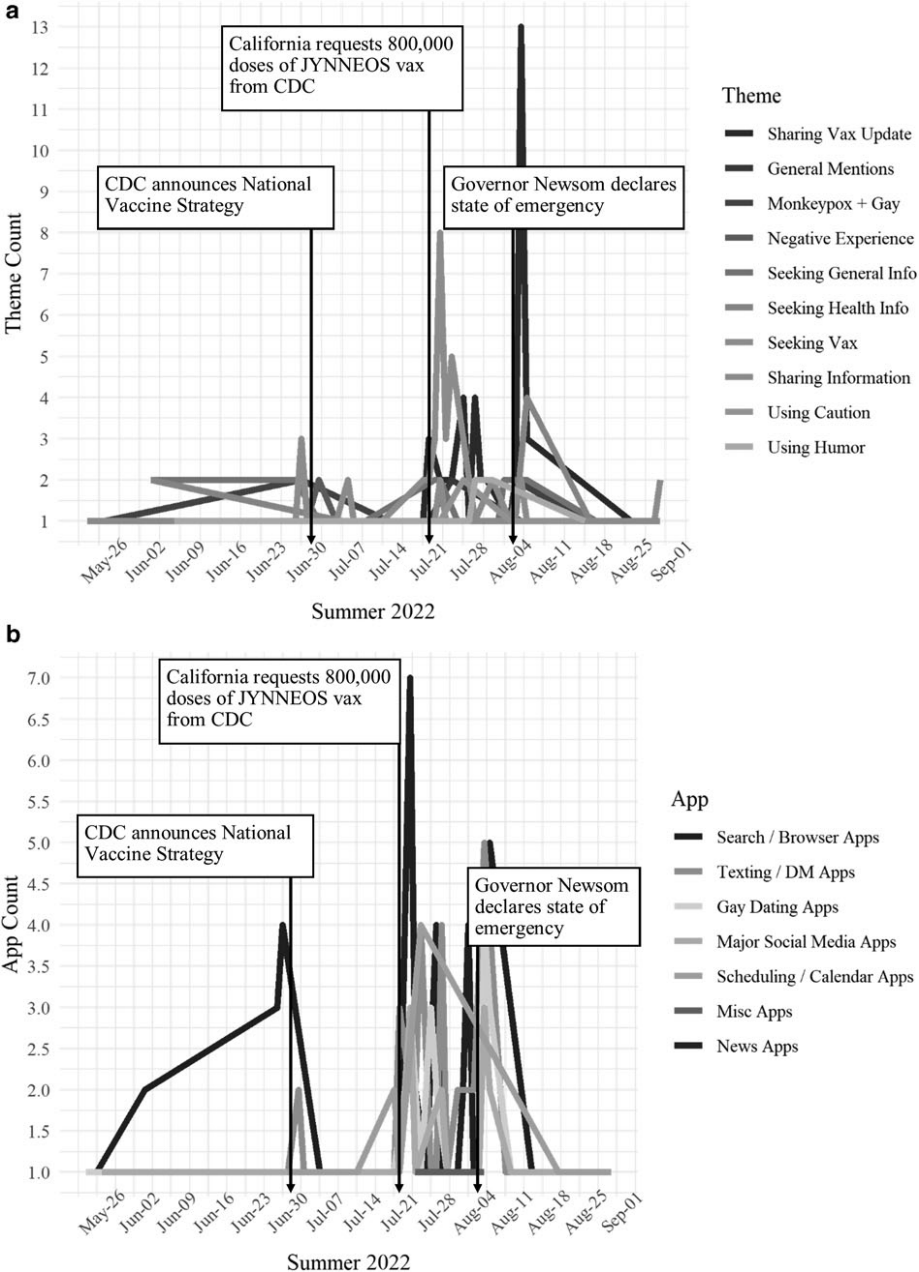
Monkeypox communication themes and channels among gay, bisexual, and other men who have sex with men. **(a)** How the frequency of individual mpox themes changed over time and **(b)** how this sample of GBMSM used apps to communicate about mpox over time. Major news developments are from Governor Newsom's^18^ Proclamation of a State of Emergency: CDC announced national vaccine strategy on June 28, 2022, California requested 800,000 doses of JYNNEOS vaccine on July 19, 2022, and Governor Newsom declared a state of emergency on August 1, 2022. App, smartphone applications; CDC, Centers for Disease Control and Prevention; DM, direct messaging.

Figure 1a depicts how the frequency of themes changed over time, overlayed with major news events in California.^18^ Of note is (1) the increase in searches for vaccines after California requested more vaccines from the Centers for Disease Control and Prevention (CDC) and (2) the surge in sharing vaccine updates with social and sexual networks after California Governor Gavin Newsom declared mpox a public health emergency (August 1, 2022).

Participants used a variety of communication channels (i.e., smartphone apps) to discuss mpox. Many GBMSM used search apps (34.5%; e.g., Firefox), texting and direct messaging apps (26.4%; e.g., Facebook Messenger). They discussed mpox on gay dating apps (14.9%; e.g., Grindr) and major social media apps (14.9%; e.g., Twitter). GBMSM mpox communication was least common on scheduling and calendar (6.9%), miscellaneous (1.7%; e.g., Microsoft Outlook), and news (0.6%) apps.

Figure 1b shows how the frequency of app use changed over time, overlayed with major news events in California.^18^ Notable observations include (1) a decrease in mpox searches on web browsers around the time the CDC announced its national vaccine strategy and (2) a surge in mpox searches on web browsers, mpox communication through text messaging services, and mpox discussions on gay dating apps after the California Health Department requested 800,000 doses of the JYNNEOS vaccine from the CDC.

## Discussion

This article analyzed how GBMSM used digital app-based technology to communicate about mpox during the 2022 outbreak. Notably, GBMSM's interest in mpox vaccination suggests a health-focused community response, which both contrasts the homonegative rhetoric blaming the mpox outbreak on GBMSM^19–21^ and mirrors the cautious, proactive, and health-focused behavior observed among other samples of GBMSM responding to mpox^22^ and among GBMSM during the COVID-19 pandemic.^10,13,23^ Results also suggest that this sample of GBMSM rapidly communicated about mpox to push back against homonegative rhetoric, to direct their peers to health information, and to modify their approaches to sexual networking.

The findings have implications for future digital interventions for viral risk reduction. Because GBMSM are concerned with vaccination, gay dating apps could partner with public health departments to offer geotargeted information on mpox vaccine availability, especially if additional outbreaks or variants emerge. Consistent with other digital interventions,^14^ public health officials also could partner with computational social scientists to build automated systems that extract meaningful insights from smartphone text data to inform health promotion messaging in real time.

Because communication themes and app use changed over time and seemed to be timed with major news events, public health departments and GBMSM-serving community-based organizations should launch health-oriented social media campaigns around major news events to promote and harness the health-focused communication already in place within GBMSM communities.

Some commentators have expressed concerns that the official U.S. federal response to mpox was inadequate, delayed, and poorly implemented.^24^ Nevertheless, mpox was brought under control relatively quickly in the United States.^25^ As suggested by our findings, it is possible that this success in controlling the U.S. outbreak was driven by the rapid health-focused communication within GBMSM networks. Network-based interventions to reduce infectious diseases, such as HIV, among GBMSM are gaining traction in public health research,^26^ but it is unclear whether steps are being taken to incorporate GBMSM communication networks into a national strategy for emergent diseases such as mpox.

In this study, GBMSM rapidly communicated about mpox shortly after the reported outbreak, communicated during periods of major news reports, and communicated risk mitigation information. These findings suggest that communication networks to reduce the impact of emergent infectious diseases among GBMSM are already present, and can be leveraged to promote effective mitigation strategies during future outbreaks.

However, before public health officials incorporate smartphone text into prevention strategies and digital interventions, how and what kinds of text data are obtained must be considered from an ethical perspective. If national organizations collect smartphone text data through an app, such as the CDC mobile app,^27^ or aggregate text data across apps (e.g., pulling text data collected by patient portals), then existing technologies would need to be modified to collect these data while stipulations about who has access to the data are created.

Privacy and third-party sharing policies would not only need to prioritize GBMSM's safety and confidentiality, but also center GBMSM's perspectives (e.g., not selling data to third parties, restricting data access to researchers)^28^ during development. Clear policies on data ownership, data deletion, and data auditing also would need to be developed. In addition, GBMSM would want the ability to determine from which apps text data are sent to public health officials (e.g., turning off data collection from private messaging apps).

It is also unclear, currently, how and to what extent these text data would be anonymized (e.g., removing the names of loved ones). Thus, although GBMSM's communication about mpox and other infectious diseases^10,13,23^ could be harnessed to promote personal and public health, several important ethical issues need to be resolved and additional research must occur before this possibility becomes reality.

### Limitations

There are limitations to this study. First, nearly all participants were from Los Angeles, California, thus limiting generalizability, especially considering that GBMSM living in rural areas use different mpox risk-reduction strategies.^29^ More research with nonurban non-U.S. GBMSM is needed to understand whether GBMSM use digital technology to communicate about infectious diseases in ways similar to this sample. Second, although examining temporal variation in mpox themes and communication channels seemed to be timed with major mpox-related news events, this study did not test and does not claim causality between news events and changes in GBMSM's mpox communication.

Mpox communication changes among this sample could be due to other variables, such as increased awareness of mpox during the summer of 2022. Longitudinal studies using larger text-based data sets (e.g., social media) and advanced methods (e.g., machine learning)—which enable causal inference^30^—are needed to infer causality between news events and GBMSM's response to mpox and other disease outbreaks. Finally, because the text data were sent over the internet to our servers, data loss during transit may have occurred, possibly resulting in data availability bias.

## Conclusion

This is the first study to examine the role of smartphone apps in facilitating a community-driven response to the mpox outbreak among GBMSM. The findings revealed that GBMSM primarily used search and browser, texting, and gay dating apps to share vaccine updates, seek mpox vaccination, find general mpox-related information, share information with other GBMSM, and discuss links between mpox and gay culture. Changes in communication themes and channels appeared to be responsive to major news events. These findings point toward the possibility of using text data in public health interventions to prevent infectious diseases by leveraging GBMSM communication networks.
